# Antagonism of the Azoles to Olorofim and Cross-Resistance Are Governed by Linked Transcriptional Networks in Aspergillus fumigatus

**DOI:** 10.1128/mbio.02215-22

**Published:** 2022-10-26

**Authors:** Norman van Rhijn, Sam Hemmings, Isabelle S. R. Storer, Clara Valero, Hajer Bin Shuraym, Gustavo H. Goldman, Fabio Gsaller, Jorge Amich, Michael J. Bromley

**Affiliations:** a Manchester Fungal Infection Group, Division of Evolution, Infection, and Genomics, Faculty of Biology, Medicine and Health, University of Manchester, Manchester, United Kingdom; b Antimicrobial Resistance Network, University of Manchester, Manchester, United Kingdom; c Faculdade de Ciências Farmacêuticas de Ribeirão Preto, Departamento de Ciências Farmacêuticas, Universidade de São Paulo, Ribeirão Preto, Brazil; d Institute of Molecular Biology/Biocenter, Innsbruck Medical University, Innsbruck, Austria; e Mycology Reference Laboratory, National Centre for Microbiology, Instituto de Salud Carlos III (ISCIII), Majadahonda, Madrid, Spain; University of British Columbia

**Keywords:** *Aspergillus fumigatus*, olorofim, orotomide, antimicrobial resistance, antifungal, transcription factor, aspergillosis, antagonism, metabolism, metabolic rewiring

## Abstract

Aspergillosis, in its various manifestations, is a major cause of morbidity and mortality. Very few classes of antifungal drugs have been approved for clinical use to treat these diseases and resistance to the first-line therapeutic class, the triazoles are increasing. A new class of antifungals that target pyrimidine biosynthesis, the orotomides, are currently in development with the first compound in this class, olorofim in late-stage clinical trials. In this study, we identified an antagonistic action of the triazoles on the action of olorofim. We showed that this antagonism was the result of an azole-induced upregulation of the pyrimidine biosynthesis pathway. Intriguingly, we showed that loss of function in the higher order transcription factor, HapB a member of the heterotrimeric HapB/C/E (CBC) complex or the regulator of nitrogen metabolic genes AreA, led to cross-resistance to both the azoles and olorofim, indicating that factors that govern resistance were under common regulatory control. However, the loss of azole-induced antagonism required decoupling of the pyrimidine biosynthetic pathway in a manner independent of the action of a single transcription factor. Our study provided evidence for complex transcriptional crosstalk between the pyrimidine and ergosterol biosynthetic pathways.

## INTRODUCTION

Invasive and chronic forms of aspergillosis affect over 3 million people resulting in more than 300 thousand deaths per year (1). Only three classes of antifungals are currently available to treat aspergillosis, with triazoles used as first-line therapy in most centers (2). Resistance to the azoles is rising, which is linked to the use of triazole compounds in agriculture and horticulture (3, 4). It is predicted that more resistant strains of A. fumigatus will be seen as azole use and will be expanded to combat climate change-associated increases in fungal crop damage (5). The development of novel classes of antifungals will be a key component in addressing the emerging resistance problem. Fortunately, several drugs represent novel classes of antifungals currently in development for the treatment of invasive aspergillosis (IA), including ibrexafungerp, which has recently (2021) gained FDA approval for the treatment of vulvovaginal candidiasis, and fosmanogepix, which targets glycosylphosphatidylinositol (GPI) anchor biosynthesis and olorofim (phase 3) (6). Olorofim (formerly known as F901318 and under development by F2G, Ltd.) is of particular interest because, like fosmanogepix, it has a novel mechanism of action that has not been exploited clinically (7). Because olorofim is orally bioavailable, it presents a realistic alternative to azoles for the long-term treatment of chronic and allergic infections and especially resistant infections (8). Moreover, it could potentially be used in combination therapy strategies to suppress the emergence of resistance.

Olorofim acts by inhibiting the enzyme dihydroorotate dehydrogenase (DHODH), encoded by the *pyrE* gene in A. fumigatus, which is a crucial enzyme within the pyrimidine biosynthesis pathway and is, thus, required for both DNA and RNA synthesis (7). Structural and biochemical analysis of dihydroorotate dehydrogenase (DHODH) suggests olorofim competes with CoQ to bind to DHODH, preventing the oxidation of dihydroorotate to orotate. DHODHs are grouped into 2 classes according to sequence similarity and subcellular localization. Both mammals and most fungi have class 2 DHODH, which is bound to the inner mitochondrial membrane (9). The human DHODH only shares a 30% protein sequence identity with the A. fumigatus DHODH, and olorofim has been demonstrated to be >2,200-fold more potent against the A. fumigatus enzyme (7). Inhibition of the pyrimidine biosynthesis pathway by olorofim prevents the germination of A. fumigatus conidia and causes hyphae to undergo morphological changes (10). Prolonged exposure of germlings and vegetative hyphae to olorofim also causes extensive isotropic expansion that is then followed by cell lysis (11).

Olorofim is effective against Coccidioides immitis, *Scedosporium* spp., Madurella mycetomatis, Lomentospora prolificans, and several Aspergillus species (12–18). However, olorofim has reduced activity against Fusarium solani species complex and Fusarium dimerum and is inactive against Mucorales (19). Olorofim is also effective against triazole-resistant A. fumigatus isolates and cryptic Aspergillus species (20, 21). In several murine models of aspergillosis, scedosporiosis, and lomentosporiosis, olorofim treatment significantly reduced fungal burden and mortality (15, 22). A recent study suggests that levels of resistance to olorofim in a collection of clinical isolates of A. fumigatus are low. Only 1 of 976 clinical isolates exhibited preexisting olorofim resistance caused by a single nucleotide polymorphism (SNP) in the *pyrE* gene (23).

In this study, we identified a concerning antagonistic effect of the triazoles on the action of olorofim in A. fumigatus. We showed that this antagonistic effect was governed by an azole-induced upregulation of the pyrimidine biosynthetic pathway. However, this did not appear to be regulated by the action of a single transcription factor. Through screening the collection of aspergillus fumigatus nulls (COFUN) A. fumigatus transcription factor null mutant library, we identified four transcription factors that regulated susceptibility to olorofim (24). Existing published literature and our phenotypic and transcriptomic data revealed these transcription factors regulated genes involved in processes immediately upstream of the pyrimidine biosynthesis pathway. Notably, two transcription factor null mutants, Δ*hapB* and Δ*areA*, had elevated MICs to olorofim and were resistant to the azole class of antifungals, highlighting potential routes to cross-resistance.

## RESULTS

### Azoles were antagonistic to the action of olorofim in a manner consistent with azole-mediated upregulation of the pyrimidine biosynthetic pathway.

**FIG 1:**
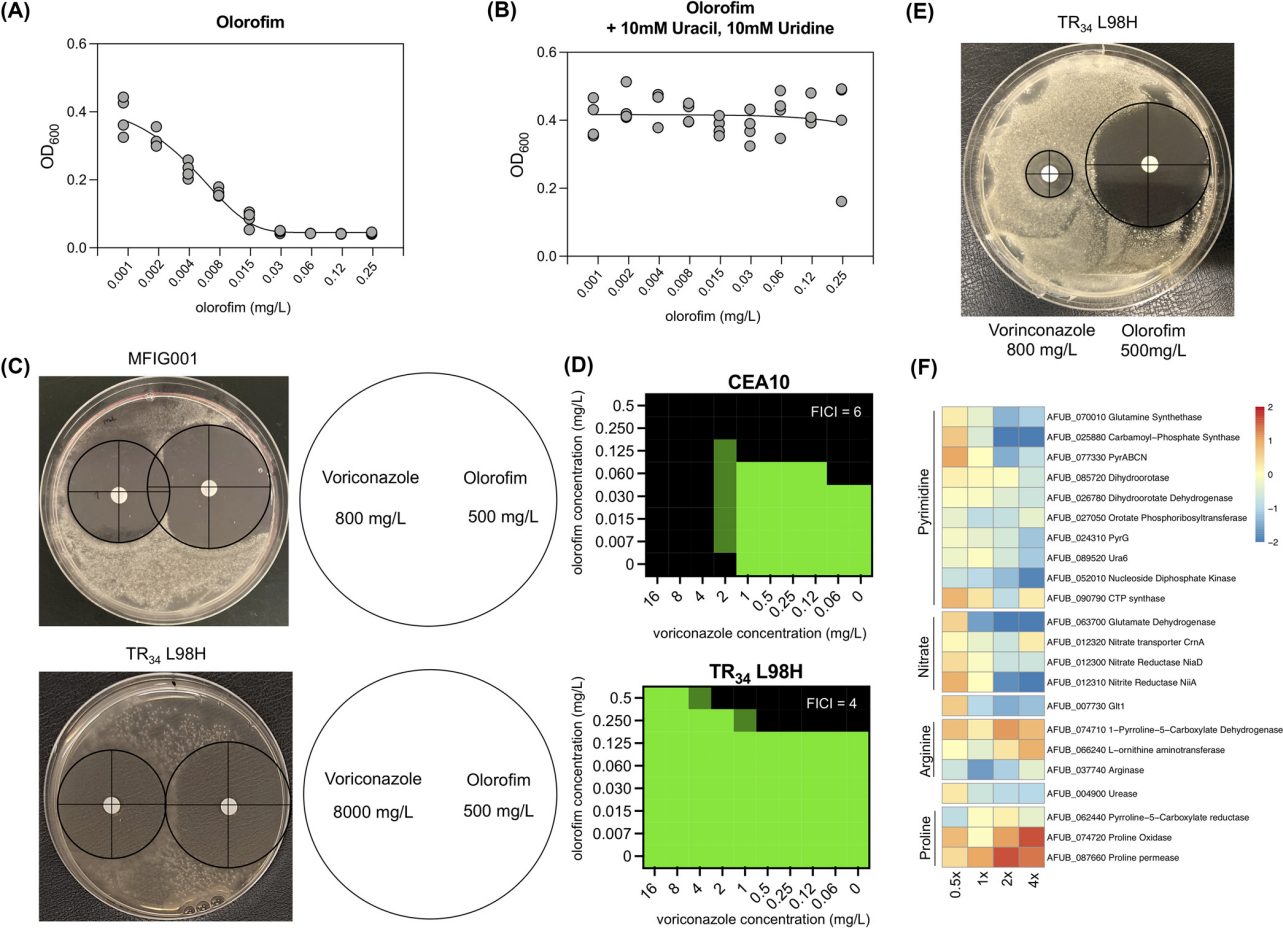
Antagonism of the azoles to olorofim. (A) Broth dilution assay of olorofim on A. fumigatus MFIG001 to olorofim, following EUCAST methodology and measured by OD_600_ (*n* = 4). (B) Addition of 10 mM uracil and 10 mM uridine reverses the action of olorofim on A. fumigatus MFIG001 (*n* = 3). (C) Antagonism on a solid RPMI 1640 plate inoculated with A. fumigatus isolates. Voriconazole (800 mg/L) was inoculated on the disk on the left and olorofim (500 mg/L) on the disk on the right. The disk assay for TR34 L98H contained 8000 mg/L voriconazole to obtain a halo of equal size to MFIG001. (D) Checkerboard assay (*n* = 3) for CEA10 and the azole resistant TR34 L98H isolate to voriconazole and olorofim. Growth was normalized to RPM-1640 without any antifungal drug. Green denotes full growth, and black denotes no observed growth. (E) Disk assay on solid RPMI 1640 at 800 mg/L voriconazole and 500 mg/L olorofim for the TR34 L98H strain. (F) Dose-response RNA-seq upon itraconazole exposure (0.5× MIC to 4× MIC). The expression of genes of the pyrimidine pathway and upstream pathways were differentially upregulated only in sub-MICs of itraconazole.

To standardize assays throughout our experiments, the MIC of olorofim against Aspergillus fumigatus MFIG001 was determined. The MIC was defined as the minimum concentration of olorofim at which no germination from Aspergillus spores was observed. Microscopic evaluation revealed the MIC of olorofim to be 0.06 mg/L for A. fumigatus MFIG001, consistent with previous findings of other A. fumigatus isolates (20). The effect of olorofim on the growth of A. fumigatus was further evaluated by measuring the optical density of the plates used to determine the MIC (Fig. 1A). The maximal growth observed (optical density at 600 nm [OD_600_] = 0.39) and absence of growth (OD_600_ = 0.04) was separated by a 64-fold difference in drug concentration, showing the effect of olorofim is progressive over a long range of concentrations until achieving total growth inhibition. This was in stark contrast to the inhibitory effects of the azoles on A. fumigatus, where the difference between maximal and minimal growth typically occurred over a drug concentration not exceeding 8-fold (Fig. S1). Because this range is broad, we considered it useful to measure the concentration at which growth was inhibited by 50% (referred to as IC_50_ (25) to distinguish from MIC_50_, which is a MIC determination made of populations). For MFIG001, the IC_50_ for olorofim was 0.0057 mg/L, whereas for itraconazole it was 0.21 mg/L. Because olorofim inhibited pyrimidine biosynthesis, it would be expected that the action of the drug would be fully reversed by supplementing the medium with an excess of exogenous pyrimidines (7). To confirm that the growth inhibition was due to directly targeting the pyrimidine biosynthesis pathway, the MIC was determined with the addition of 10 mM uridine and 10 mM uracil (Fig. 1B). Under these conditions, there was no observed reduction in A. fumigatus growth, and at all olorofim concentrations, the median OD_600_ did not fall below control levels, indicating that there are no significant off-target effects of this drug.

10.1128/mbio.02215-22.1 FIG S1 Determination of IC_50_ for itraconazole. MIC to olorofim in RPMI 1640 was determined according to EUCAST methodology for A. fumigatus MFIG001. OD_600_ was measured after 48 h to determine growth quantitatively. Download FIG S1, JPG file, 0.02 MB. Copyright © 2022 van Rhijn et al. 2022 van Rhijn et al. https://creativecommons.org/licenses/by/4.0/ This content is distributed under the terms of the Creative Commons Attribution 4.0 International license.

Resistance to the clinical azoles has become a global problem that is being addressed in multiple centers by using combination therapy with either an echinocandin or amphotericin B. If approved for use, olorofim may be used in the same way. We, therefore, investigated the potential interaction in activity between the triazoles, voriconazole, itraconazole, and olorofim against CEA10, MFIG001, and a TR34 L98H azole-resistant isolate generated in the MFIG001 background (26). To our surprise, given the distinct mechanisms of action of the orotomides and the azoles, we observed a clear unidirectional antagonism by the azoles on olorofim in both liquid cultures using a checkerboard assay (fractional inhibitory concentration index [FICI] was 6 and 4 for CEA10 and TR34 L98H, respectively) resulting in a 4-fold increase in MIC to olorofim and solid medium as evidenced by the growth induced by voriconazole within the halo expected for olorofim (Fig. 1C and D). Interestingly, we did not see the same antagonism between olorofim and manogepix, another late-stage antifungal compound (Fig. S2). Significantly, the antagonism of the azoles to olorofim was also observed under nongrowth inhibitory concentrations of voriconazole for the TR34 L98H azole-resistant isolate, showing that this antagonistic response was independent of the azole antifungal activity (Fig. 1E).

10.1128/mbio.02215-22.2 FIG S2 Antagonism between manogepix and olorofim. Antagonism was assessed and quantified (*n* = 6) between manogepix and olorofim. A synergy between these two novel antifungals was observed. Download FIG S2, JPG file, 0.3 MB. Copyright © 2022 van Rhijn et al. 2022 van Rhijn et al. https://creativecommons.org/licenses/by/4.0/ This content is distributed under the terms of the Creative Commons Attribution 4.0 International license.

To gain an understanding of the potential mechanisms driving this antagonism, we evaluated transcriptomic data for A. fumigatus MFIG001 exposed to increasing concentrations of itraconazole (Fig. 1F). As expected, the ergosterol biosynthetic pathway was differentially regulated throughout itraconazole concentrations. At sub-MIC levels of itraconazole, we observed a significant upregulation of genes in the pyrimidine biosynthetic pathway and those pathways that generate its precursors (Data Set S1). Most strikingly, the nitrate assimilation pathway, *glt1*, which encoded glutamate synthase, and the first three steps in the pyrimidine pathway that utilized glutamate (encoded by *glnA*-AFUB_070010, *pyrD*-AFUB_085720, and *pyrABCN-AFUB_077330* and its orthologues *AFUB_025880* and *AFUB_054340*) were upregulated in sub-MIC levels of itraconazole (Fig. 1E). Interestingly, many of these genes were downregulated in supra-MICs of itraconazole, suggesting metabolic arrest (27). This led us to hypothesize that both the pyrimidine pathway and ergosterol biosynthesis pathways were potentially coregulated.

10.1128/mbio.02215-22.9 DATA SET S1 DESeq2 results of the response of A. fumigatus A1160p+ to olorofim. Download Data Set S1, XLSX file, 2.7 MB. Copyright © 2022 van Rhijn et al. 2022 van Rhijn et al. https://creativecommons.org/licenses/by/4.0/ This content is distributed under the terms of the Creative Commons Attribution 4.0 International license.

### Deletion of HapB, AreA, DevR, and AcdX changed olorofim susceptibility.

**FIG 2:**
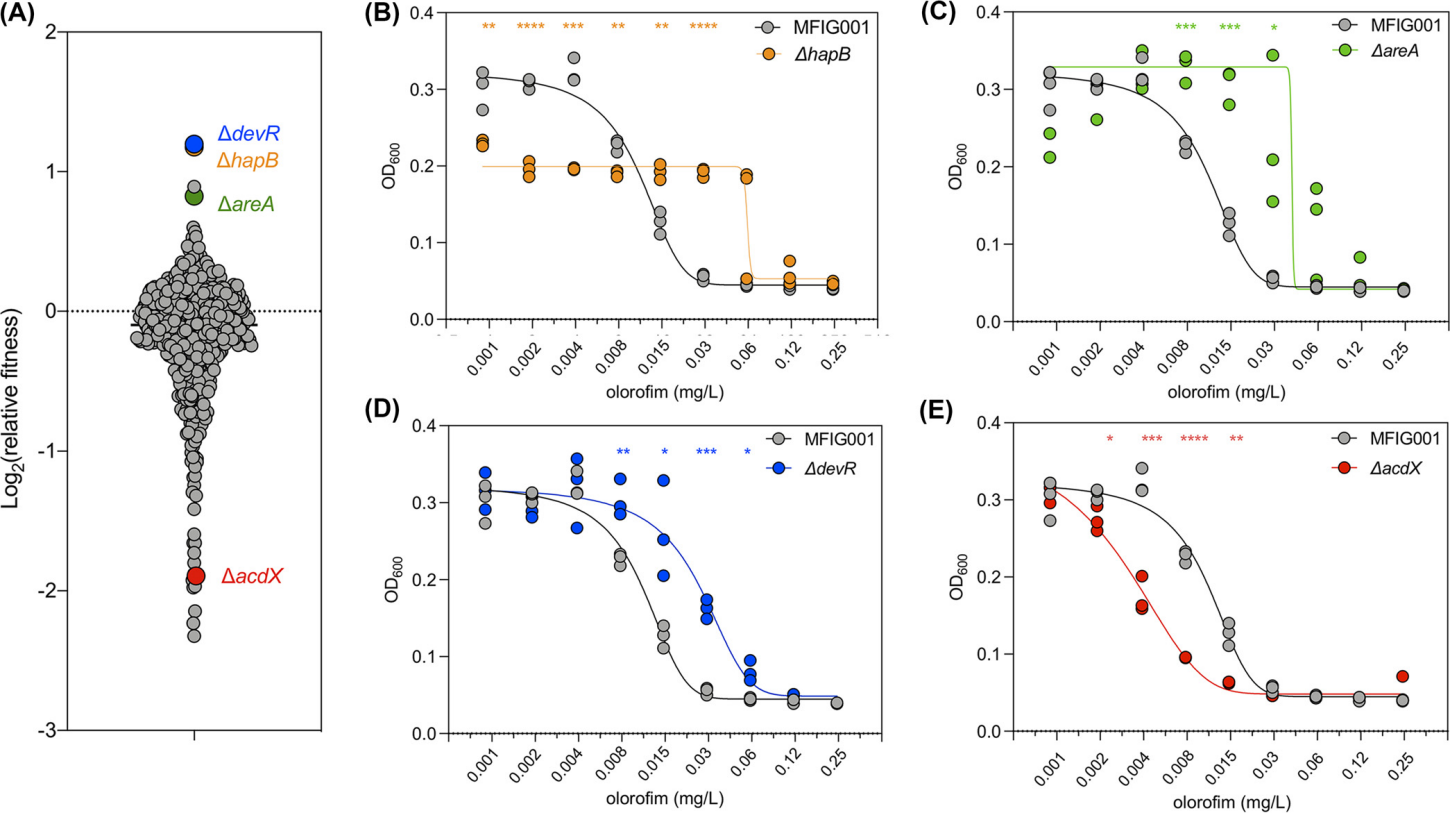
Olorofim susceptibility screening of the COFUN transcription factor knockout library. (A) The relative fitness of each strain was assessed by normalizing it to fitness in nondrug conditions (*n* = 3). TF null mutants that were of particular interest are highlighted. (B to E) Broth dilution assay of olorofim on the TF null mutants, (B) for Δ*hapB*, (C) for Δ*areA*, (D) for Δ*devR*, (E) for Δ*acdX*, as determined by OD_600_ (*n* = 3). Statistical difference was assessed by a two-way ANOVA with Sidaks multiple-comparison test (*, *P* < 0.05; **, *P* < 0.01; ***, *P* < 0.001; ***, *P* < 0.001).

As we observed antagonism between the azoles and olorofim, and coregulation of those pathways upon azole exposure, we hypothesized that both pathways may be regulated by the same transcription factors. To assess this coregulation and identify novel transcriptional regulators associated with differential olorofim susceptibility and azole antagonism, the COFUN transcription factor knockout (TFKO) library was screened against olorofim at a concentration that reduces the growth of the isogenic wild-type isolate (MFIG001) by about 20% (0.002 mg/L). At this concentration, we were able to identify strains that have the potential to be resistant or hypersensitive (Fig. 2A) while utilizing resource-limiting levels of a drug.

Three transcription factor null mutants (Δ*areA*, Δ*hapB*, and Δ*devR*) showed reproducible increased relative fitness in the presence of olorofim and elevated MICs compared to MFIG001 (Fig. 2B to D). Remarkably, two of these mutants (Δ*areA* and Δ*hapB*) were also resistant to the azole class of antifungals (24). Loss of AreA, a transcription factor that had a global role in activating the expression of genes involved in nitrogen acquisition and processing (28), or loss of HapB, which along with HapC and HapE comprised the CCAAT binding complex (CBC) (29) resulted in a 2-fold increase in MIC to olorofim compared to the isotype control MFIG001. IC_50_ values for these strains were impacted much more with 50% growth inhibition reached at 0.04 mg/L (4-fold increase) for Δ*areA* and 0.07 mg/L (8-fold increase) for Δ*hapB* (Fig. 2B and C). This simultaneous decrease in azole and olorofim susceptibility suggests a higher-level regulatory link between ergosterol biosynthesis and pyrimidine biosynthesis. DevR is a bHLH transcription factor involved in sporulation and melanin biosynthesis (30). The Δ*devR* mutant showed a significant reduction in susceptibility to olorofim at concentrations ranging from 0.008 mg/L to 0.06 mg/L (MIC) and had an IC_50_ of 0.025 mg/L (Fig. 2D). Although the MIC for this strain increased to >0.125 mg/L most spores did not germinate at this concentration.

One isolate (ΔAFUB_056620, Δ*acdX*) showed a reproducible significant increase in sensitivity to olorofim and had a MIC of 0.03 mg/L and an IC_50_ of 0.006 mg/L, 2-fold lower than A. fumigatus MFIG001 (Fig. 2E). The *acdX* gene encoded a 612 amino acid transcription factor that contained six WD40 repeat units but no other functional domains, as shown by a simple modular architecture research tool (SMART) domain search. A reciprocal BLAST search of the AFUB_056620 protein sequence found a match to the Saccharomyces cerevisiae transcription factor Spt8. However, the proteins only shared 44% identity of the entire protein sequence. In S. cerevisiae, Spt8 formed part of the SAGA (Spt-Ada-Gcn5-acetyltransferase) complex (31), which is known to act as a transcriptional activator under several stress conditions. While the orthologue of AcdX in other fungi generally contained six WD40 domains, in species like N. crassa and A. terreus, only five domains are present. However, the significance of this is unclear. In A. nidulans, AcdX has been described to be functional in the SAGA complex and is involved in repressing genes in acetate metabolism, and has a regulatory role in the proline metabolic pathway (32).

### Transcription factor mutants with altered susceptibility to olorofim had defects in nitrogen assimilation.

**FIG 3:**
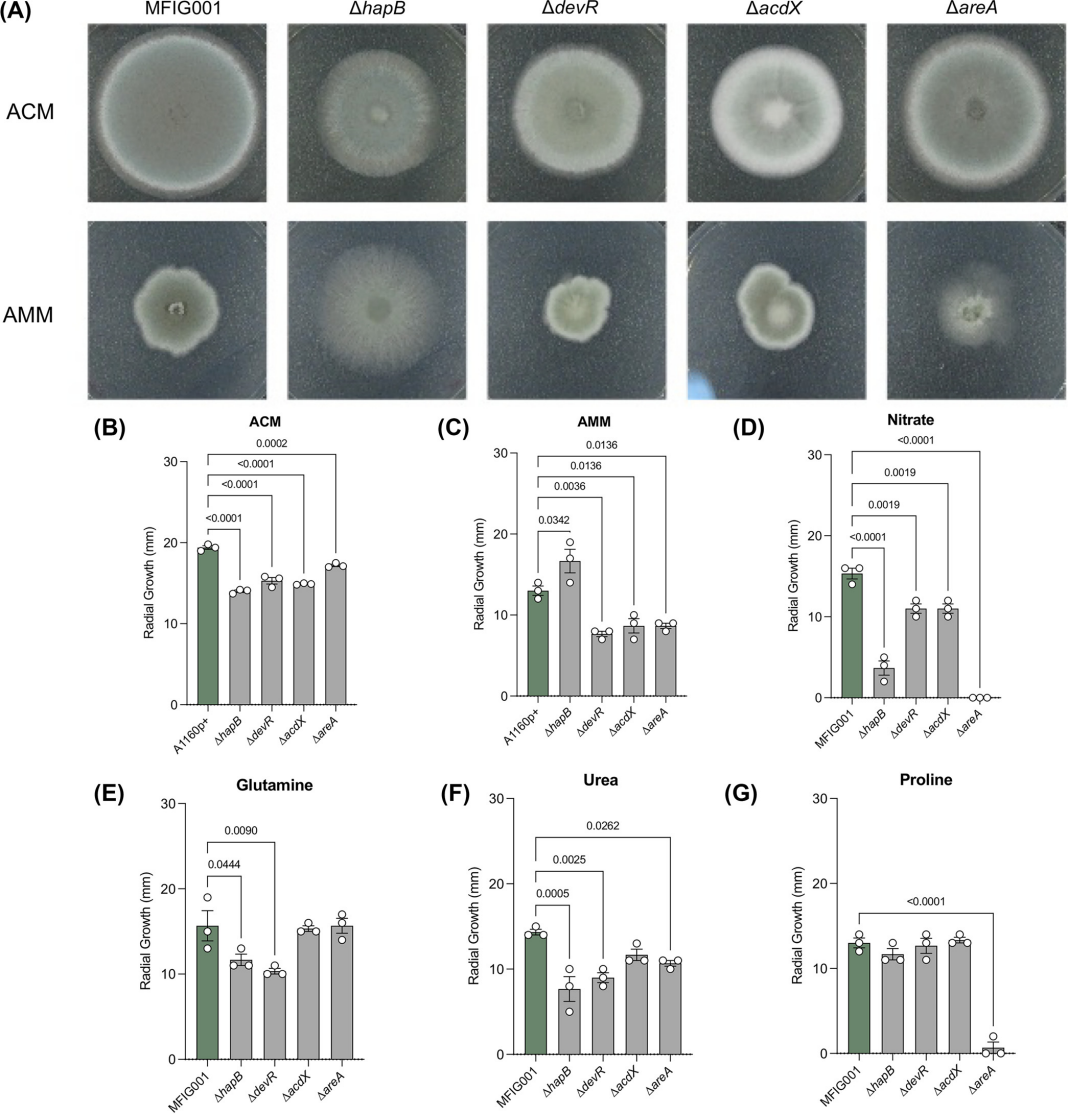
Phenotypic evaluation of TF null mutants. (A) 500 spores of TF null mutants and MFIG001 were spotted on Aspergillus complete medium and Aspergillus minimal medium and incubated for 48 h at 37°C. (B and C) Radial growth of TF null mutants and MFIG001 on ACM (B) and AMM (C), after 72 h at 37°C (*n* = 3) (D to G) TF null mutants spotted on AMM supplemented with 10 mM sodium nitrate (D), 10 mM l-glutamine (E), 10 mM urea (F), or 10 mM l-proline (G) (*n* = 3) Statistical difference was assessed by two-way ANOVA with Dunn’s correction (*P* < 0.05 are shown).

Further phenotypic analysis of the null mutants with differential susceptibility to olorofim revealed that all had differential growth on Aspergillus complete medium (ACM) (Fig. 3A and B) and Aspergillus minimal medium (AMM), which contained ammonium tartrate as a nitrogen source (Fig. 3A and C). The *hapB*, *devR*, *areA*, and *acdX* null mutants showed a reduction of radial growth on ACM of 28%, 22%, 12%, and 24%, respectively, compared to the isotype control (*P* < 0.05). On AMM, the *hapB* mutant showed an increase in radial growth (58%). However, colony growth was more diffuse than the isotype strain (Fig. 3A and C). Because olorofim inhibited DHODH, which acted within the pyrimidine biosynthetic pathway, we hypothesized that these growth defects could be reflecting an alteration in the abundance of precursors of this pathway. As expected for a strain that was unable to initiate a nitrate assimilation response, when ammonium tartrate was substituted with nitrate, the Δ*areA* isolate was unable to grow. Similarly, the growth defect for the Δ*hapB* isolate was exacerbated in this media. The growth defects of the other transcription factor null mutants were not rescued (Fig. 3D and Fig. S3). Glutamine substitution rescued the growth rate defects of Δ*acdX* and Δ*areA*, although significant phenotypic growth defects were still present even after supplementation (Fig. 3E). Similarly, urea almost completely rescued Δ*acdX* and proline fully rescued Δ*hapB*, Δ*devR*, and Δ*acdX* (Fig. 3F and G). Taken together, these results showed that these transcription factor null mutants had defects in nitrogen utilization that, given its connection with the pyrimidine pathway, could be linked to olorofim susceptibility.

10.1128/mbio.02215-22.3 FIG S3 Images of nitrogen spot tests of TF null mutants. Growth of TF null mutants and wild-type was assessed on AMM supplemented with 50 mM ammonium tartrate, 10 mM sodium nitrate, 10 mM l-glutamine, 10 mM urea, or 10 mM l-proline (*n* = 3). Images were taken after 72 h at 37°C. Download FIG S3, JPG file, 0.8 MB. Copyright © 2022 van Rhijn et al. 2022 van Rhijn et al. https://creativecommons.org/licenses/by/4.0/ This content is distributed under the terms of the Creative Commons Attribution 4.0 International license.

### Changes in susceptibility to olorofim in Δ*devR* and Δ*acdX* mutants were caused by opposing regulation of pathways preceding pyrimidine biosynthesis.

**FIG 4:**
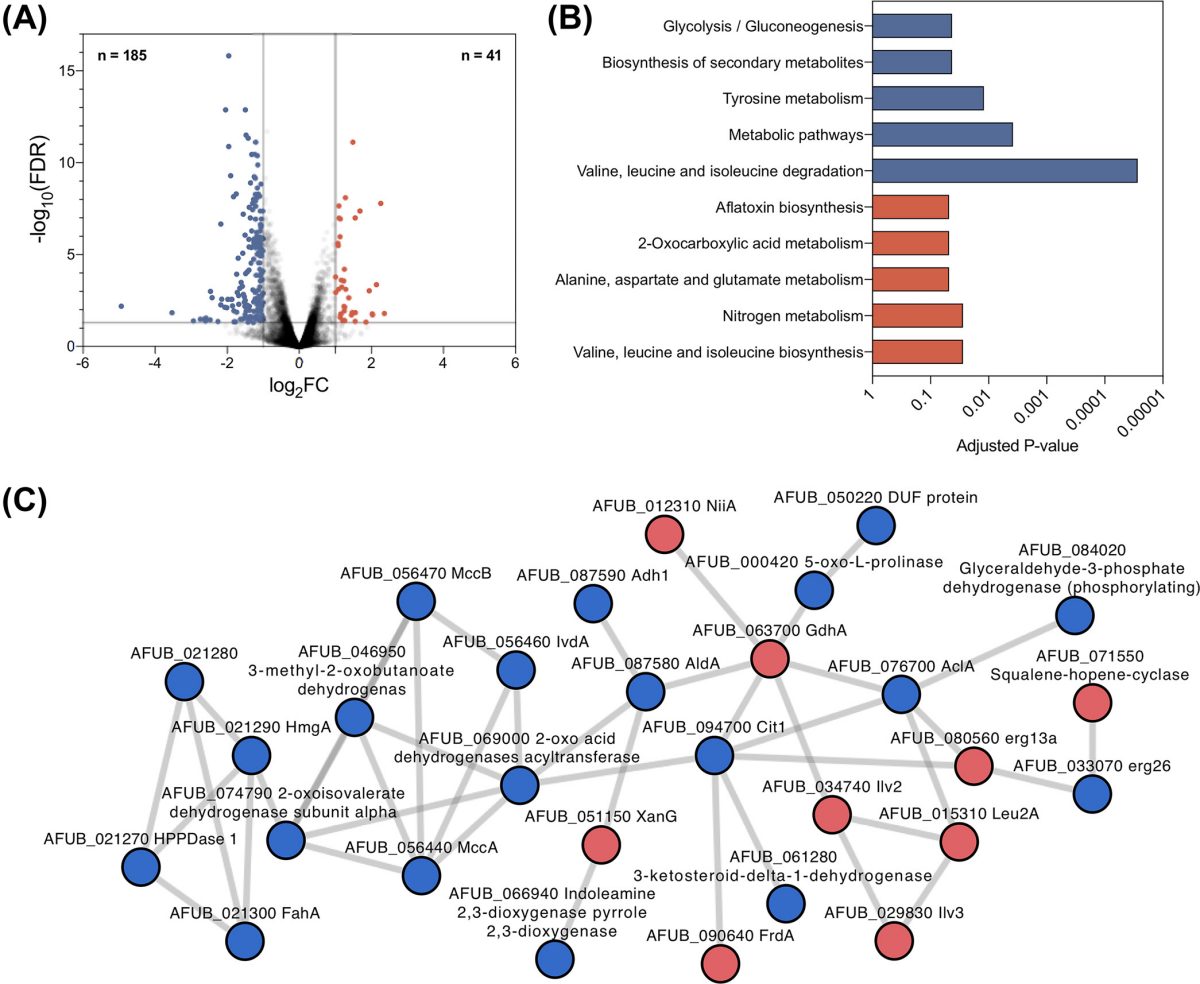
Transcriptomics of MFIG001 to olorofim. (A) Volcano plot of RNA-seq of A. fumigatus MFIG001 exposed to olorofim. In total, 185 genes (blue dots) and 41 genes (red dots) were considered downregulated and upregulated, respectively (>2-fold differentially regulated; *P* < 0.05). (B) KEGG pathways were enriched within differentially regulated genes, blue categories denote those associated with downregulated genes, and red denotes those with upregulated genes. (C) Interactions of proteins involved in response to olorofim as determined by StringsDB. Proteins derived from upregulated transcripts are in red and downregulated in blue.

To facilitate our understanding of how these transcription factors were functioning to alter olorofim sensitivity, we performed a whole transcriptome analysis. Upon olorofim exposure (1× MIC) for 1 h, a modest 41 genes and 185 genes were upregulated and downregulated with log_2_ > 1 (Fig. 4A) in our isotype-type strain, respectively. We expected that several genes in the immediate pyrimidine biosynthesis pathway would be upregulated but only the gene encoding the multifunctional carbamoyl-phosphate synthase/aspartate carbamoyltransferase (PyrABCN, AFUB_077330) enzyme, which is upstream of DHODH and converted carbamoyl-P to N-carbamoyl-l-aspartate, was upregulated by log_2_ > 1 (Data Set S1). Instead, genes associated with pathways that synthesize precursors of the pyrimidine biosynthetic pathway were identified, including oxaloacetate metabolism and glutamate biosynthesis (Fig. 4B and C; Data Set S1). Genes associated with tyrosine metabolism, secondary metabolite biosynthesis, glycolysis/gluconeogenesis, and valine, leucine, and isoleucine degradation were enriched among downregulated genes (Fig. 4B). A search tool for the retrieval of interacting genes/proteins (STRINGS) analysis of differentially regulated genes showed an interconnected network of genes involved in ergosterol biosynthesis, the TCA cycle and nitrogen metabolism (Fig. 4C).

**FIG 5:**
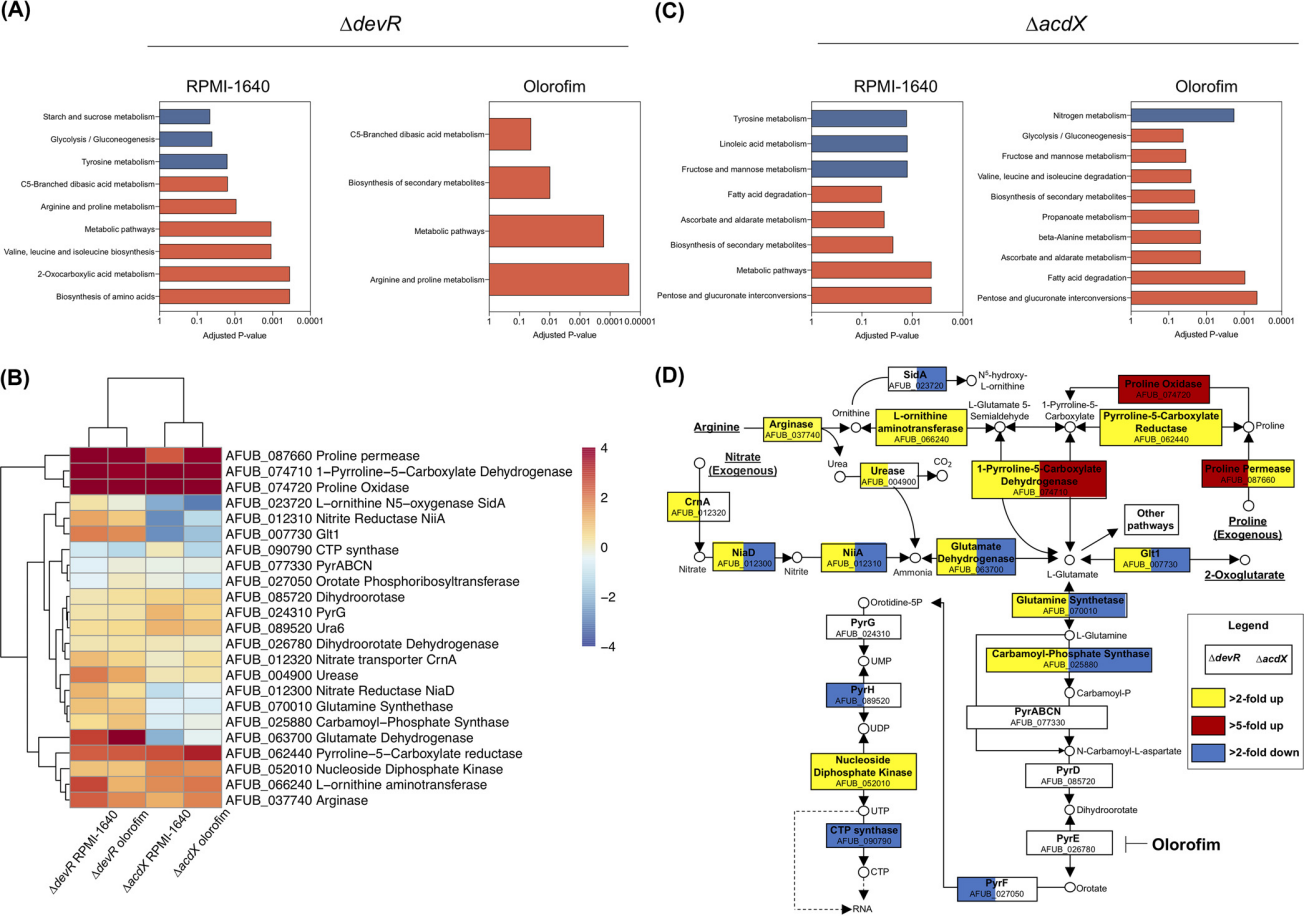
RNA-seq analysis of Δ*devR* and Δ*acdX* exposed to olorofim. (A) KEGG pathways enriched for downregulated (blue) or upregulated (red) genes in RPMI 1640 or upon olorofim exposure for Δ*devR* compared to A. fumigatus A1160p+. (B) Heatmap of genes involved in the pyrimidine pathway and components upstream of this pathway. (C) KEGG pathways enriched for downregulated (blue) or upregulated (red) genes in RPMI 1640 or upon olorofim exposure for Δ*acdX* compared to A. fumigatus MFIG001. (D) Detailed analysis of genes involved in pathways upstream of and including the pyrimidine pathway. The target of olorofim, DHODH, is highlighted. Blue denotes more than 1-fold downregulated, yellow denotes more than 1-fold upregulated, and red denotes more than 5-fold upregulated. The right of each box is associated with Δ*acdX* and the left with Δ*devR*.

To characterize the basis of differential olorofim susceptibility in the Δ*devR* and Δ*acdX* mutants, the transcriptomes of these two mutants were compared to the wild-type (Data Set S2). In the absence of olorofim, 510 and 137 genes were, respectively, downregulated and upregulated in the Δ*devR* isolate while 212 were downregulated and 194 upregulated upon olorofim exposure. In the absence of olorofim, notable enriched functional categories included downregulation of genes involved in tyrosine metabolism and an upregulation of genes involved in the biosynthesis of branched-chain amino acids and metabolism of arginine and proline, the latter of which was also seen under olorofim exposure (Fig. 5A). A detailed pathway analysis under olorofim challenge of genes involved in the conversion of metabolites toward l-glutamate and through to orotate revealed that proline uptake and degradation were upregulated in the *devR* null mutant (Fig. 5B and D). Other pathways that contribute to orotate precursors were also significantly upregulated, notably the nitrate assimilation pathway ((NAP [*crnA*, *niaD*, *niiA*]), and glutamate, glutamine, and carbomoyl-P synthesis). Pathways that competed with orotidine biosynthesis for l-glutamate were not differentially regulated in any of the assessed mutants (Data Set S2). Our transcriptional data, therefore, suggested that nitrogen metabolism was probably altered in this strain in ways that favored the generation of precursors for orotate biosynthesis and, hence, could explain the reduced sensitivity of *devR* null mutant to olorofim.

10.1128/mbio.02215-22.10 DATA SET S2 DESeq2 results of the response of A. fumigatus Δ*acdX* and Δ*devR* to olorofim. Download Data Set S2, XLSX file, 10.8 MB. Copyright © 2022 van Rhijn et al. 2022 van Rhijn et al. https://creativecommons.org/licenses/by/4.0/ This content is distributed under the terms of the Creative Commons Attribution 4.0 International license.

The transcriptome of the olorofim hypersensitive Δ*acdX* mutant also revealed that proline and arginine metabolism were upregulated compared to the wild-type, but genes involved in the NAP and glutamate, glutamine, and carbomyl-P synthesis pathways were downregulated suggesting that AcdX and DevR have directly opposing functions on these linked pathways (Fig. 5C and D) and providing further evidence to suggest that regulation of these pathways was important for olorofim sensitivity.

Our transcriptomic data and the phenotype of the null mutants led us to assess the effect of pyrimidine pathway precursors on olorofim susceptibility in the transcription factor null mutants. A. fumigatus will utilize glutamine as a preferential nitrogen source even in the presence of other nitrogen-containing compounds, such as nitrate, because pathways that process these precursors are repressed (33, 34). Intriguingly, however, when nitrate was added to the glutamine containing RPMI 1640, the sensitivity of A. fumigatus to olorofim increased indicating that even in the presence of preferential nitrogen sources, nitrate could initiate an adaptive response (Fig. S4). In the olorofim resistant, nitrate nonutilizing strain Δ*areA*, the addition of nitrate to RPMI reduced susceptibility levels back to that observed for the wild-type. For the Δ*devR* isolate, where the nitrate assimilation pathway, as well as all other pathways leading to pyrimidine biosynthesis, were upregulated, the addition of nitrate did not reduce olorofim susceptibility. The olorofim hypersensitive *acdX* null was the most impacted by changes in nitrogen sources and counterintuitively given the downregulation of the NAP in this strain, the addition of nitrate reduced olorofim susceptibility. These data, combined with results from our transcriptomic analysis suggested that modification of environmental nitrogen sources and or dysregulation of nitrogen metabolism directly impacted changes in olorofim sensitivity.

10.1128/mbio.02215-22.4 FIG S4 The effect of nitrogen source on olorofim susceptibility. MICs according to EUCAST methodology in RPMI-1640 supplemented with either 20 mM arginine, 10 mM nitrate, 20 mM proline, or 50 mM glutamine. The addition of nitrate changed Olorofim susceptibility by 2-fold for all strains except Δ*devR*. Download FIG S4, JPG file, 0.5 MB. Copyright © 2022 van Rhijn et al. 2022 van Rhijn et al. https://creativecommons.org/licenses/by/4.0/ This content is distributed under the terms of the Creative Commons Attribution 4.0 International license.

### Azole-mediated antagonism of olorofim was linked to dysregulation of pyrimidine precursor pathways but was not mediated by transcription factors that governed drug resistance.

**FIG 6:**
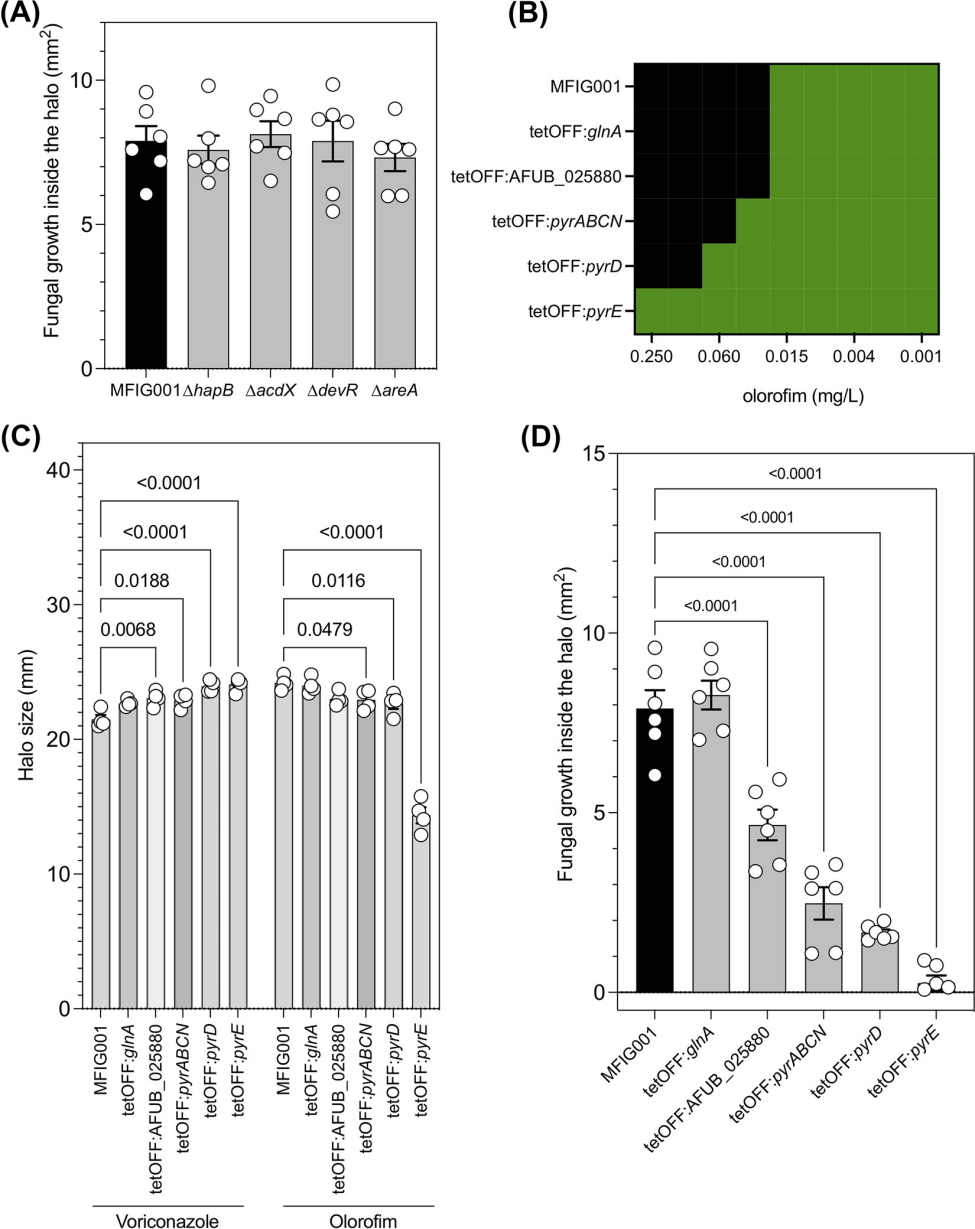
Antagonism between olorofim and the azoles through dysregulation of the pyrimidine pathway. (A) Antagonism for the TF mutants with differential susceptibility to olorofim (*n* = 6) (B) Broth microdilution assay by EUCAST methodology to olorofim for MFIG001 and the generated tetOFF mutants in the pyrimidine pathway (*n* = 3). (C) The halo size for the generated tetOFF mutants in the pyrimidine pathways to voriconazole and olorofim (*n* = 4). Statistical significance was assessed using a one-way ANOVA with Dunnett’s correction (*P* < 0.05 are shown). (D) Antagonism for the tetOFF mutants within the pyrimidine pathway (*n* = 6). Statistical significance was assessed using a one-way ANOVA with Dunnett’s correction (*P* < 0.05 are shown).

Next, we assessed if the transcription factor null mutants with differential susceptibility to olorofim retained antagonism by voriconazole. To our surprise, antagonism, as determined by the area of growth inside the olorofim halo, was not affected in these mutants (Fig. 6A and Fig. S5). This indicated that antagonism was more complex and potentially required multiple regulatory factors. This led us to hypothesize that we could affect antagonism by unlinking the pyrimidine pathway from the transcriptional effect of the addition of sub-MICs of azole. Therefore, we replaced the promoters of *glnA* (AFUB_070010), *pyrABCN* (AFUB_077330), and its paralogues AFUB_025880, *pyrD* (AFUB_085720) and *pyrE* (AFUB_026780) with the doxycycline-regulatable promoter (tetOFF). As expected, replacing the native promoter of *pyrE* with the highly expressing and inducible tetOFF promoter increased the expression of *pyrE* (c. 25-fold; Fig. S6A) and a dramatic decrease in susceptibility to olorofim when assessed by broth microdilution (Fig. 6B). In keeping with our hypothesis that genes upstream of *pyrE* were also important in mediating olorofim susceptibility, modest but reproducible decreases in susceptibility were also observed when the promoters of either *pyrABCN* or *pyrD* were replaced. Next, we assessed the susceptibility of these mutants on a solid medium using a disk assay, by measuring the diameter of the inhibition halo. Strikingly under the same conditions, the susceptibility of the strains to the azoles increased, suggesting that strains may be hypersensitive to the azoles if resistance to olorofim was induced by upregulation of this pathway (Fig. 6C).

10.1128/mbio.02215-22.5 FIG S5 Antagonism of the azoles to olorofim for TF mutants. Representative images of TF mutants with differential susceptibility to olorofim tested for antagonism between voriconazole (800 mg/L) and olorofim (500 mg/L). Download FIG S5, JPG file, 0.9 MB. Copyright © 2022 van Rhijn et al. 2022 van Rhijn et al. https://creativecommons.org/licenses/by/4.0/ This content is distributed under the terms of the Creative Commons Attribution 4.0 International license.

10.1128/mbio.02215-22.6 FIG S6 Overexpression of *pyrE* by inserting the tetOFF cassette. (A) The *pyrE* gene was overexpressed by inserting the tetOFF cassette as a promoter system. Expression was measured by qPCR (*n* = 3) without doxycycline and could be reduced by the addition of doxycycline (50 μg/mL). (B) The halo size for tetOFF:*pyrE* at different doxycycline concentrations. (C) The area inside the halo at different concentrations of doxycycline (*n* = 6) for the tetOFF:*pyrE* mutant. Download FIG S6, JPG file, 0.1 MB. Copyright © 2022 van Rhijn et al. 2022 van Rhijn et al. https://creativecommons.org/licenses/by/4.0/ This content is distributed under the terms of the Creative Commons Attribution 4.0 International license.

To ensure there was no significant impact on changing the susceptibility of the azoles in our assessment of antagonism in our plate assay, doxycycline levels were titrated to ensure the halo induced by olorofim and voriconazole was almost identical to that of MFIG001 (Fig. S6B). Consistent with our hypothesis that azole-induced antagonism was mediated by the pyrimidine biosynthesis pathway, antagonism (as measured by fungal growth within the halo or via a checkerboard assay) was reduced in a stepwise manner within genes of the pyrimidine pathway and completely ablated in the tetOFF:*pyrE* regardless of the amount of doxycycline used (Fig. 6D; Fig. S6C and S7).

10.1128/mbio.02215-22.7 FIG S7 Checkerboard assay of tetOFF mutants to olorofim and voriconazole. Checkerboard assays (*n* = 2) for tetOFF mutants to voriconazole and olorofim. Growth was normalized to RPMI 1640 without any antifungal drug. Green equals full growth, black no observed growth. Calculated FICI are shown in the upper right corner of each assay. Download FIG S7, JPG file, 0.1 MB. Copyright © 2022 van Rhijn et al. 2022 van Rhijn et al. https://creativecommons.org/licenses/by/4.0/ This content is distributed under the terms of the Creative Commons Attribution 4.0 International license.

In conclusion, we identified high-level coordination of the regulation of azole and orotomide resistance, seemingly caused by crosstalk between the control of the ergosterol and pyrimidine biosynthetic pathways. These pathways were induced in the presence of the azoles, resulting in an antagonistic effect on the novel DHODH inhibitor olorofim.

## DISCUSSION

Olorofim is a novel antifungal, currently in phase 3 clinical trials. It has a broad spectrum of activity against most molds and acts by inhibiting the pyrimidine biosynthetic pathway through disruption of DHODH activity (7). Our preliminary analysis of the inhibitory effects of olorofim revealed that the MIC and the IC_50_ were separated over a relatively large concentration range (5-fold). This contrasted with what has been observed with itraconazole and other azoles where this concentration spread was typically 2-fold. The clinical implication of this finding remains unclear. However, olorofim will likely support clearance of infection at doses well below the MIC. At these lower concentrations, however, drug exposure will be imparting selective pressure and has the potential to induce the production of mutagenic precursors that may drive the emergence of resistance as has been shown for several antibiotics (35). As with other anti-infectives that act by inhibiting a single biological target there is clear potential for the emergence of resistance. Understanding these mechanisms will provide a framework for the development of diagnostics to detect resistance rapidly in the clinic.

Our previous survey of itraconazole sensitivity in the A. fumigatus COFUN transcription factor knockout library (36) revealed 6 null mutants that had decreased sensitivity (ranging from 4 to 6-fold increase in MIC compared to the isogenic control) and 6 had increased sensitivity (4 to 8-fold decrease in MIC) to itraconazole. Here, our screen revealed that only 1 mutant (Δ*acdX*) showed increased sensitivity while 3 showed decreased sensitivity (Δ*hapB*, Δ*devR*, and Δ*areA*) to olorofim. The changes in sensitivity in these isolates were less extreme than seen for the azoles, indicating that the frequency of olorofim resistance may be lower than that seen for itraconazole. Indeed, this hypothesis was supported by a recent study that revealed the frequency of olorofim resistance was variable between strains ranging from 1.3 × 10^−7^ to 6.9 × 10^−9^, while for itraconazole resistance occurred at an order of magnitude higher (1.2 × 10^−6^ and 3.3 × 10^−8^) (23). It is unsurprising, given the mechanism of action of olorofim, that the transcription factors that we identified in this screen either have well-defined roles in regulating nitrogen utilization or have been linked to this function in our study.

What is remarkable, however, given the distinct mechanisms of actions of the two compound classes, loss of function of either AreA and HapB resulted in cross-class resistance to both the azoles and orotomides. HapB is a member of the heterotrimeric CCAAT-binding complex (CBC) and, alongside HapC and HapE, regulates the expression of over a third of the genome (26), including several genes involved in ergosterol biosynthesis. The *hapB* null displayed the highest levels of resistance to olorofim and was able to germinate at 0.12 mg/L, which is 8-fold higher than the parental isolate but within the concentration range needed for clinical utility. In A. nidulans, AreA is a positive regulator of many genes that are required for utilization of nitrogen sources other than glutamate or ammonia (37) with loss of function resulting in an inability to utilize among other nitrogen sources, nitrate, nitrite, uric acid, and many amino acids (38). Reassuringly, however, drug concentrations in animal models are tolerated well above the increased MIC levels of the null mutants identified in this screen. Dosing 8 mg/kg at 8 h intervals in mice resulted in peak serum levels of 2.5 to 3 mg/L (8). Olorofim coudl be tolerated at doses as high as 30 mg/kg intravenously, giving scope for higher drug levels *in vivo*, if required. In cynomolgus monkeys, a single oral dose of olorofim resulted in peak levels of 0.605 to 0.914 mg/L in serum for female and male animals, respectively (39).

Our studies have shown there is a clear unidirectional antagonism of the azoles on olorofim, mediated by azole-induced overexpression of the pyrimidine biosynthetic pathway and/or metabolic flux through this pathway. While concerning, the antagonism was only evident when relatively low levels of both drugs were used. It is interesting to note that the TR_34_ L98H isolate used in this study had reduced susceptibility to olorofim compared to the CEA10 isolate, and the antagonism drove the MIC above 0.5 mg/L. Whether this is of clinical significance remains to be determined. Interestingly, overexpression of any part of the pyrimidine biosynthetic pathway resulted in a modest increase in susceptibility of A. fumigatus to the azoles, indicating that some strains that were resistant to olorofim may be more susceptible to the azoles and highlighting that there is complex crosstalk between the ergosterol and pyrimidine biosynthetic pathways. If these drugs are to be used in combination in a clinical setting, careful evaluation of respective drug levels at the site of infection to ensure sufficient concentration of drug to avoid antagonism would be sensible. The consequences of using azoles and olorofim in combination for the treatment of strains harboring the TR_34_ L98H allele also need further evaluation.

In summary, we explored the mechanism behind olorofim susceptibility through a systematic analysis of the COFUN transcription factor null library. All the mutants we identified that had altered sensitivity to olorofim had associated defects in nitrogen metabolism. Two of these mutants, Δ*devR*, and Δ*acdX*, showed dysregulation of genes involved in metabolic pathways immediately upstream of the pyrimidine pathway, potentially leading to a differential flux of metabolites into this pathway. Importantly, we identified two transcription factors, the CBC and AreA, that regulated cross-resistance to both the azoles and olorofim. Lastly, we detected an antagonistic effect between olorofim and the azoles, which we could modulate through transcriptionally unlinking the pyrimidine pathway from upstream pathways.

## MATERIALS AND METHODS

### Fungal strains.

Conidia of Aspergillus fumigatus MFIG001 (a derivative of CEA10) and transcription factor null mutants (24, 40) were prepared by inoculating strains in vented 25 cm^2^ tissue culture flasks with Sabouraud Dextrose agar (Oxoid, Hampshire, England) and incubating at 37°C for 48 h. Spores were harvested in PBS + 0.01% Tween 20 by filtration through Miracloth. Spores were counted using a hemocytometer (Marienfeld Superior, Baden-Württemberg, Germany). To generate the TR34/L98H isolate the coding region of *cyp51A* was amplified by PCR (using primers cyp51a_fw and cyp51a_rv) from an azole-resistant clinical isolate (F10017) and transformed into a previously generated isolate that harbored the tandem repeat duplication (TR34) in the MFIG001 (26). Transformants were selected for voriconazole resistance (>16 mg/L) and validated by Sanger sequencing.

### Olorofim MIC screening.

Olorofim was a kind gift of F2G Ltd. The MIC of olorofim against A. fumigatus was assessed using the European Committee for Antimicrobial Susceptibility Testing (EUCAST) methodology (19, 41). Briefly, 2 × 10^4^ spores/mL (in 100 μL) were added to a CytoOne 96-well plate (StarLab, Brussels, Belgium) containing 1 × RPMI-1640 medium (Sigma-Aldrich, St. Louis, MO), 165 mM 3-(N-morpholino) propanesulfonic acid) (MOPS) buffer (pH 7.0), 2% glucose, with olorofim 2-fold dilution series ranging from 0.1 μg/L to 0.25 mg/L and a drug-free control (*n* = 4). Additionally, a serial dilution of olorofim containing 10 mM uracil and uridine was performed. The 96-well plates were incubated at 37°C for 48 h. The MIC was determined as the minimum drug concentration at which no germination was observed. Optical density was measured at 600 nm using a Synergy HTX Multi-Mode Microplate Reader (BioTek, Winooski, VT). In keeping with research laboratory-based definitions, but in contrast to definitions used clinically, we defined *in vitro* resistance as a strain that was less susceptible to the drug than the parental isolate (42).

### Olorofim sensitivity screening of the A. fumigatus transcription factor null mutant library.

In total, 2 × 10^4^ spores/mL from each of the 484 members of the transcription knockout library were added to 1× RPMI 1640 medium, 165 mM MOPS buffer (pH 7.0), 2% glucose in each well of a CytoOne 96-well plate with 0.002 mg/L olorofim (*n* = 4). Plates were incubated at 37°C for 48 h. Fitness was calculated by dividing the optical density of respective null mutants by the MFIG001 control. Relative fitness in olorofim was calculated by dividing fitness in olorofim with general growth fitness of the transcription factor null mutants using the same microculture conditions in 1× RPMI 1640 medium, 165 mM MOPS buffer (pH 7.0), and 2% glucose without olorofim (*n* = 4). Optical density was measured at 600 nm on a Synergy HTX Multi-Mode Microplate Reader (BioTek, Winooski, VT).

### RNA extraction.

For RNA extraction, 1 × 10^6^ spores/mL of A. fumigatus MFIG001, Δ*AFUB_056620*, and Δ*AFUB_030440* were inoculated into 50 mL of Aspergillus complete medium (ACM) (43) and incubated for 18 h at 37°C in a rotary shaker (180 rpm). Mycelia were harvested using filtration through Miracloth (Merck Millipore) and washed in 1× RPMI 1640 medium. Approximately 1 g of mycelia was added to shake flasks containing 50 mL RPMI 1640 medium, 165 mM MOPS buffer (pH 7.0), and 2% glucose and then incubated for 1 h at 37°C in a rotary shaker (180 rpm) in the presence or absence of 0.062 mg/L olorofim (*n* = 3) or the presence or absence of 0.25 mg/L, 0.5 mg/L, 1 mg/L, or 2 mg/L itraconazole (*n* = 3) and incubated for 4 h. Mycelia was filtered through Miracloth and snap-frozen using liquid nitrogen and kept at −80°C until required.

To extract RNA, 1 mL of TRIzol reagent (Sigma-Aldrich) and 710 to 1180 μm acid-washed glass beads (Sigma-Aldrich) were added to frozen mycelia and placed in a TissueLyser II (Qiagen, Hilden, Germany) for 3 min at 30 Hz. The solution was centrifuged (12,000 rpm) for 1 min at 4°C. The aqueous phase was added to 200 μL of chloroform and centrifuged (12,000 rpm) for 10 min at room temperature. The supernatant was added to 0.2 M sodium citrate, 0.3 M sodium chloride, and 25% (vol/vol) isopropanol and then left at room temperature for 10 min. This solution was centrifuged (12,000 rpm) for 15 min at 4°C. The supernatant was removed, and the pellet was washed in 70% (vol/vol) ethanol and then resuspended in RNase-free water (Thermo Fisher Scientific, Waltham, MA). RNA samples were treated with RQ1 RNase-Free DNase (Promega, Madison, WI) and purified using an RNeasy Minikit (Qiagen). RNA quality and quantity were assessed using gel electrophoresis and using a NanoDrop^TM^ 2000/2000c Spectrophotometer (Thermo Fisher Scientific). All RNA extractions were carried out in triplicate.

### Transcriptomic analysis.

RNA sequencing was carried out by the Genomic Technologies Core Facility (GTCF) at the University of Manchester. Sequencing libraries were prepared from mRNA using TruSeq Stranded mRNA assay (Illumina, San Diego, CA). Samples were sequenced on a single lane on an Illumina HiSeq2500 (Illumina). Low-quality reads of resulting fastq files were removed using FastQC and trimmed using Trimmomatic (quality >20, sliding window average of 4 bases) (44). Bowtie was used to align libraries to the A. fumigatus A1163 genome assembly GCA_000150145.1 with gene annotation from CADRE/Ensembl Fungi v24 (45). Differential expression analysis was performed using DESeq2 (46).

Functional category and gene ontology enrichment analysis was carried out using FungiFun2 2.2.8, converting genes to Af293 gene names to allow using the KEGG option (47). Genes that showed over 2-fold in differential expression and Benjamin-Hochberg FDR <0.01 underwent enrichment analysis. StringsDB analysis was performed by only, including genes with at least two connections.

### Phenotypic analysis.

For colony images, 500 spores per isolate were spotted onto solid ACM or Aspergillus minimal medium (AMM) and left to dry. Plates were incubated at 37°C for 72 h and imaged. Growth on solid AMM supplemented with different nitrogen sources (50 mM ammonium tartrate, 10 mM sodium nitrate, 10 mM l-glutamine, 10 mM urea, or 10 mM l-proline) was assessed by spotting 500 spores from each isolate (*n* = 3). Plates were incubated at 37°C for 72 h. MICs were determined using the same supplementation as the phenotypic test with a serial dilution of olorofim (ranging from 0.1 μg/L to 0.25 mg/L). The 96-well plates were incubated for 48 h at 37°C, and growth was determined by microscopic evaluation.

### Checkerboard assays.

For assessing drug combination efficacies of itraconazole and olorofim against A. fumigatus, we used a checkerboard assay similar to the EUCAST MIC testing described above. Two-fold serial dilutions of itraconazole were prepared across the *x*-axis and olorofim serial dilutions across the *y*-axis. The MIC was determined by microscopy by visually assessing the well containing the lowest drug concentration with nongerminated spores. The fractional inhibitory concentration index (FICI) was calculated as the MIC in combination divided by the MIC of individual drugs (48).

### Generation of TetOFF mutants.

The tetOFF cassette was amplified from pSK606 (49) containing 50 bp homology arms targeted to the promoter of each target gene (Table S1). These PCR products were used as a repair template for CRISPR-Cas9 mediated transformation (50) using the corresponding crRNA for each gene (Table S1). Transformants were selected using pyrithiamine (concentration) containing AMM + 1% sorbitol plates, purified twice, and validated by PCR.

10.1128/mbio.02215-22.8 TABLE S1 Oligos and crRNA used in this study. Download Table S1, XLSX file, 0.01 MB. Copyright © 2022 van Rhijn et al. 2022 van Rhijn et al. https://creativecommons.org/licenses/by/4.0/ This content is distributed under the terms of the Creative Commons Attribution 4.0 International license.

### Disk assays.

In total, 4 × 10^4^ conidia of the relevant A. fumigatus strain were evenly distributed on solidified 1× RPMI 1640 (Sigma), 165 mM MOPS buffer (pH 7.0), and 2% glucose. One 6 mm antibiotic assay disk (Whatman) was placed on the middle of the plate or two disks at a fixed distance, and 10 μL of voriconazole (800 mg/L), olorofim (500 mg/L), manogepix (250 mg/L) or H_2_O_2_ (30%) were added to each of them. The plates were incubated at 37°C for 48 h and imaged. Antagonism was measured as the area within the halo when two antifungals are combined showing fungal growth. Measurements were done using FIJI.

### Data availability.

RNA-seq data are available from ArrayExpress as an experiment with accession no. E-MTAB-10590. The differential expression output from DESeq2 is included as Data Set S1. Itraconazole RNA-seq is available from GEO with accession no. PRJNA861909.
