# Supplementary figures and images for: Antagonism of the Azoles to Olorofim and Cross-Resistance Are Governed by Linked Transcriptional Networks in Aspergillus fumigatus
Source: mBio. 2022 Oct 26;13(6):e02215-22. doi: 10.1128/mbio.02215-22 (PMC9765627; doi:10.1128/mbio.02215-22)

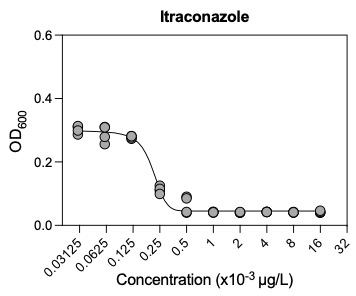

Supplement: FIG S1 [file mbio.02215-22-s0004.jpg]

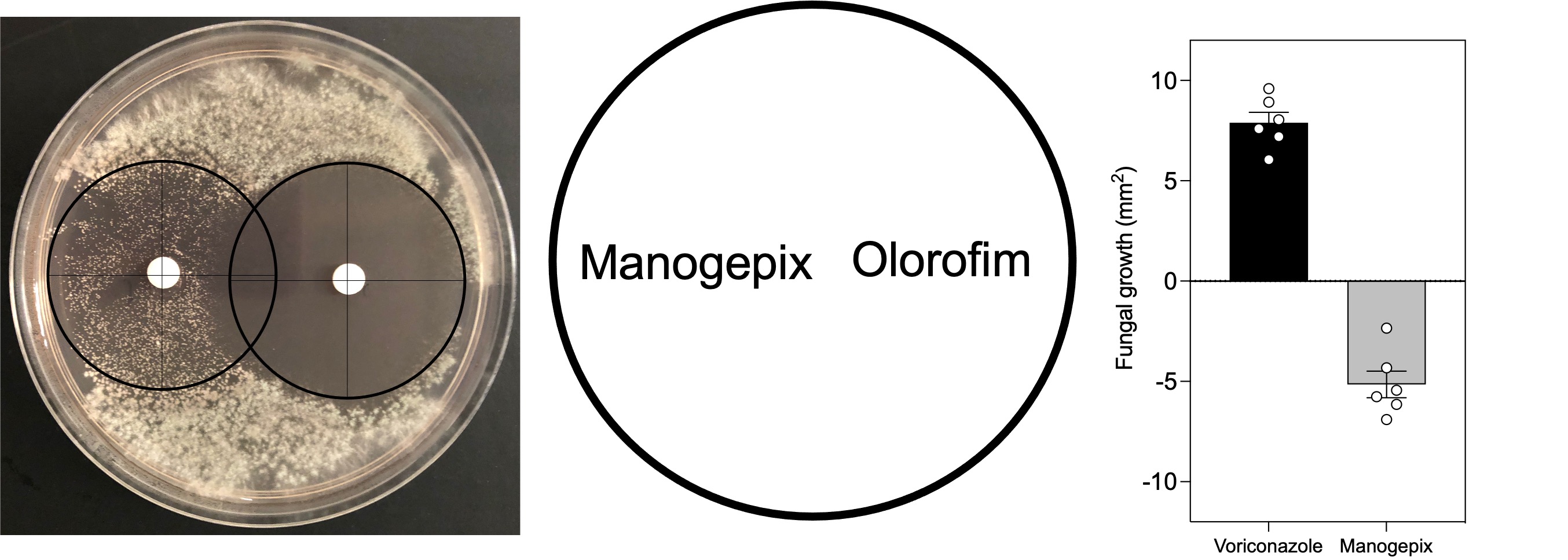

Supplement: FIG S2 [file mbio.02215-22-s0005.jpg]

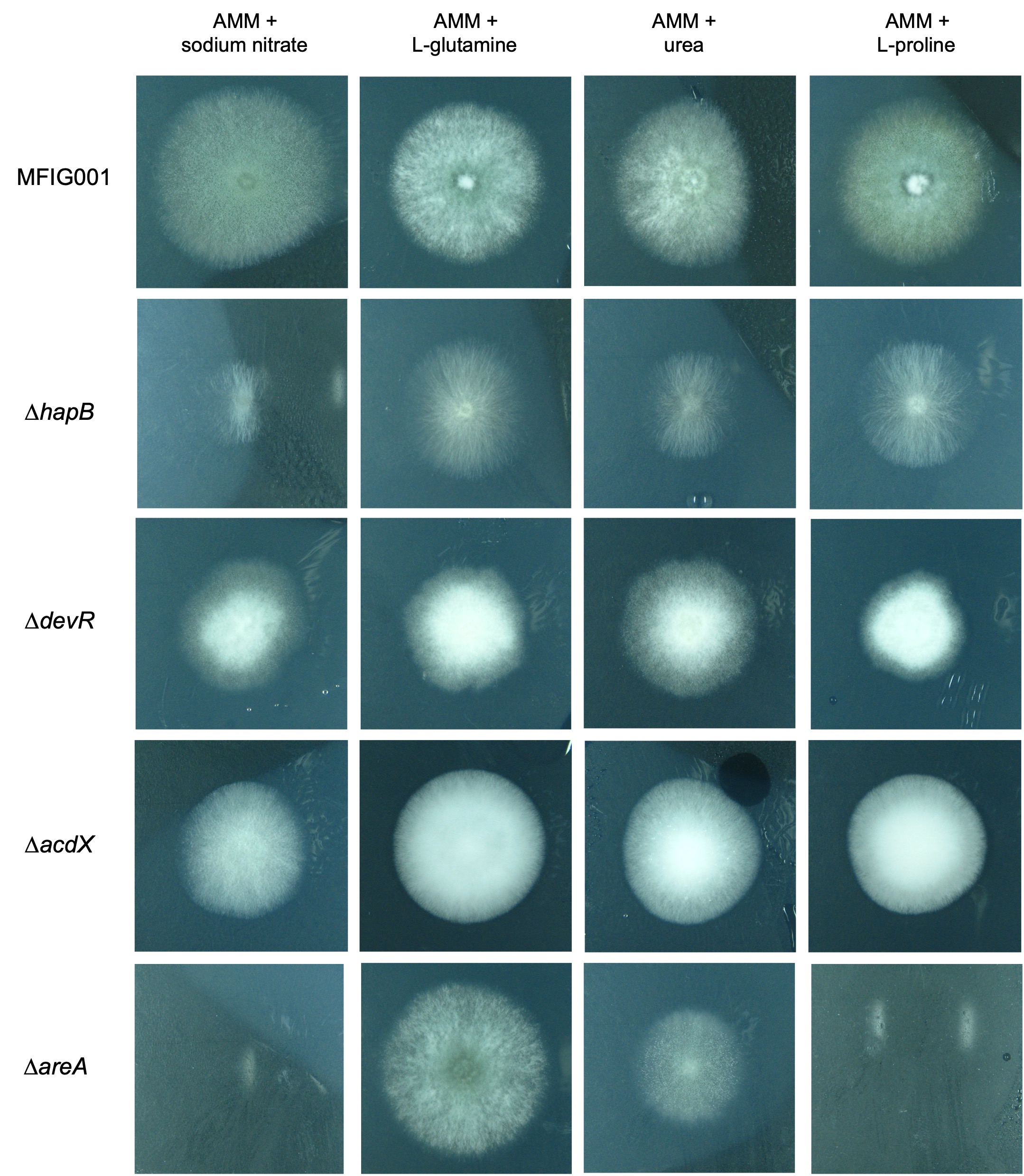

Supplement: FIG S3 [file mbio.02215-22-s0006.jpg]

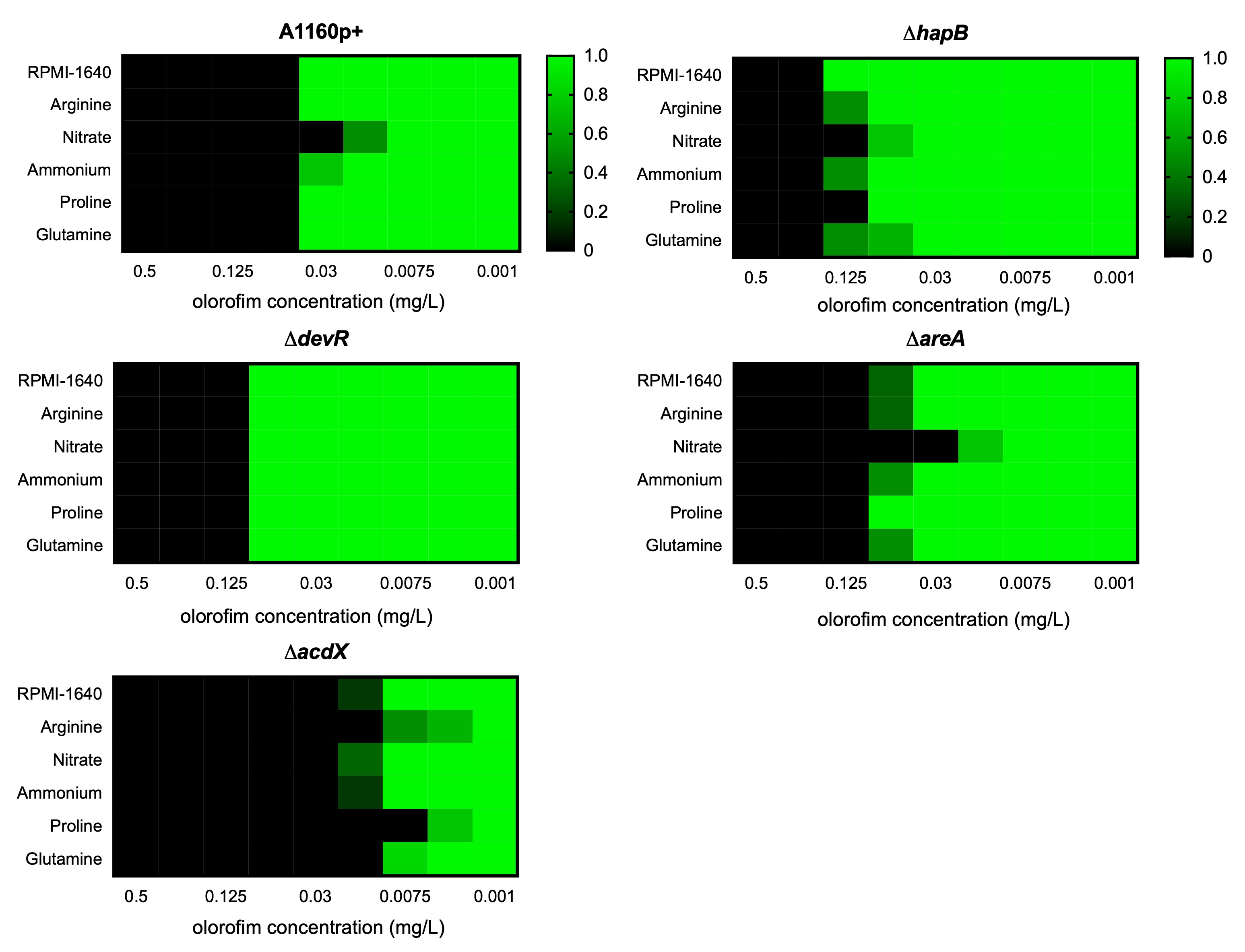

Supplement: FIG S4 [file mbio.02215-22-s0007.jpg]

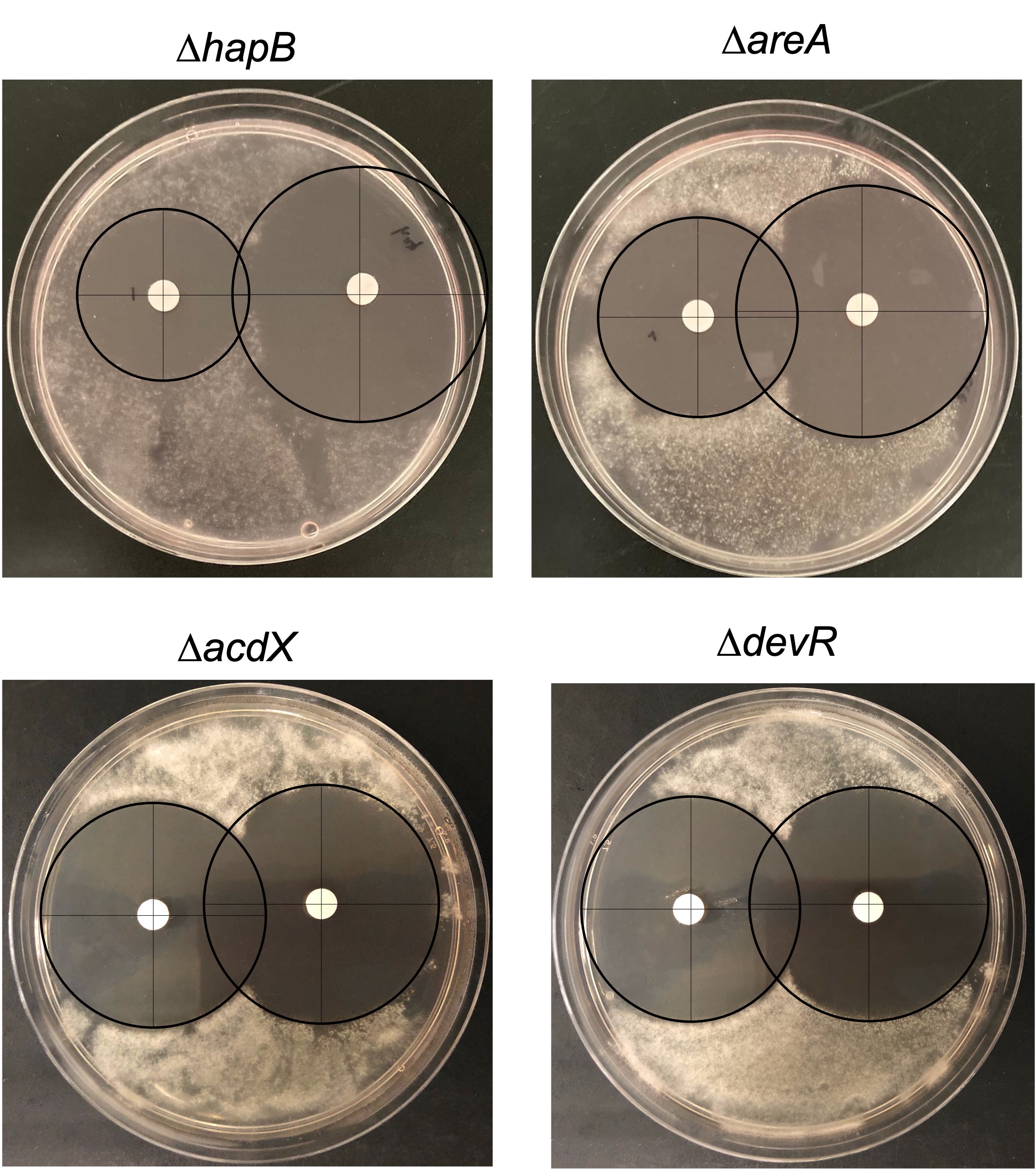

Supplement: FIG S5 [file mbio.02215-22-s0008.jpg]

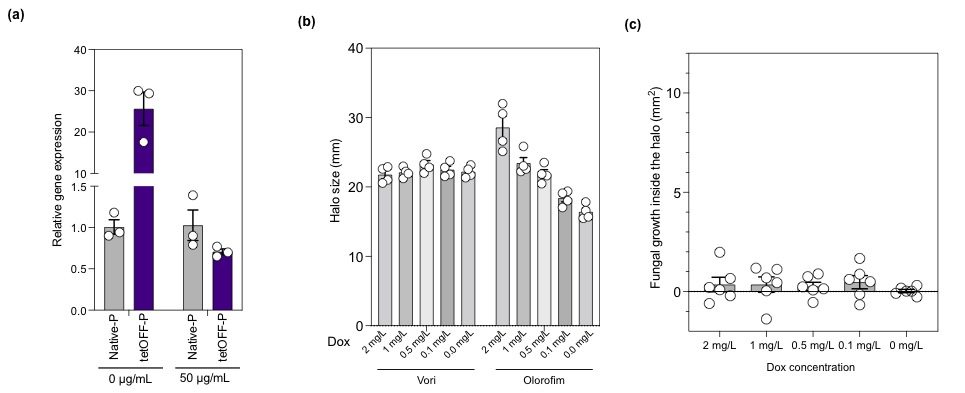

Supplement: FIG S6 [file mbio.02215-22-s0009.jpg]

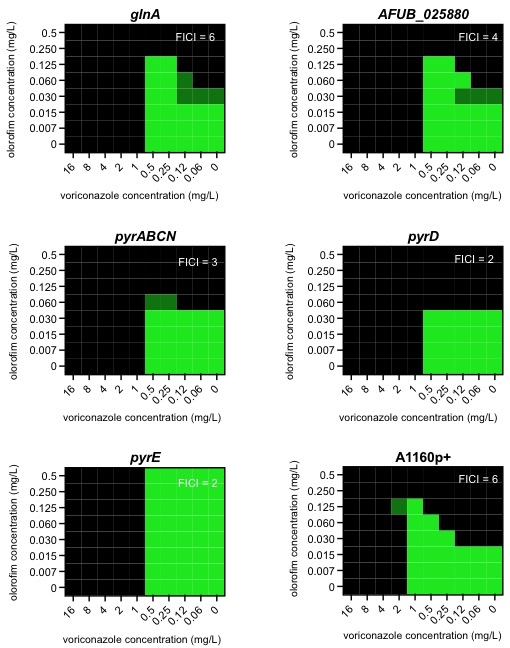

Supplement: FIG S7 [file mbio.02215-22-s0010.jpg]
